# The *ATXN1* and *TRIM31* genes are related to intelligence in an ADHD background: Evidence from a large collaborative study totaling 4,963 Subjects

**DOI:** 10.1002/ajmg.b.31149

**Published:** 2010-12-16

**Authors:** Thais S Rizzi, Alejandro Arias-Vasquez, Nanda Rommelse, Jonna Kuntsi, Richard Anney, Philip Asherson, Jan Buitelaar, Tobias Banaschewski, Richard Ebstein, Dina Ruano, Sophie Van der Sluis, Christina A Markunas, Melanie E Garrett, Allison E Ashley-Koch, Scott H Kollins, Arthur D Anastopoulos, Narelle K Hansell, Margaret J Wright, Grant W Montgomery, Nicholas G Martin, Sarah E Harris, Gail Davies, Albert Tenesa, David J Porteous, John M Starr, Ian J Deary, Beate St. Pourcain, George Davey Smith, Nicholas J Timpson, David M Evans, Michael Gill, Ana Miranda, Fernando Mulas, Robert D Oades, Herbert Roeyers, Aribert Rothenberger, Joseph Sergeant, Edmund Sonuga-Barke, Hans Christoph Steinhausen, Eric Taylor, Stephen V Faraone, Barbara Franke, Danielle Posthuma

**Affiliations:** 1 Department of Functional Genomics, CNCR, Neuroscience Campus Amsterdam,VU University and VU Medical CenterAmsterdam, the Netherlands; 2 Department of Psychiatry, Donders Institute for Brain, Cognition and Behavior, Radboud University Nijmegen Medical CenterNijmegen, the Netherlands; 3 Department of Human Genetics, Radboud University Nijmegen Medical CenterNijmegen, the Netherlands; 4 MRC Social, Genetic, and Developmental Psychiatry Centre, Institute of Psychiatry, King's College LondonLondon, United Kingdom; 5 Department of Psychiatry, Trinity Centre for Health Sciences, St. James's HospitalDublin, Ireland; 6 Department of Cognitive Neuroscience, Donders Institute for Brain, Cognition and Behavior, Radboud University Nijmegen Medical CenterNijmegen, the Netherlands; 7 Department of Child and Adolescent Psychiatry and Psychotherapy, Central Institute of Mental Health, University of HeidelbergMannheim, Germany; 8 Departments of Psychology, National University of Singapore and Hebrew UniversityJerusalem, Israel; 9 Section Medical Genomics, VUMCAmsterdam, the Netherlands; 10 Center for Human Genetics, Duke University Medical CenterDurham, North Carolina; 11 Department of Psychiatry, Duke University Medical CenterDurham, North Carolina; 12 Department of Psychology, University of North CarolinaGreensboro, North Carolina; 13 Queensland Institute of Medical ResearchBrisbane, Australia; 14 Centre for Cognitive Ageing and Cognitive Epidemiology, Medical Genetics Section, University of EdinburghEdinburgh, UK; 15 Centre for Cognitive Ageing and Cognitive Epidemiology, Department of Psychology, University of EdinburghEdinburgh, UK; 16 MRC Human Genetics Unit, The Institute of Genetics and Molecular Medicine, The University of Edinburgh, Western General HospitalEdinburgh, UK; 17 Centre for Cognitive Ageing and Cognitive Epidemiology, Geriatric Medicine Unit, University of Edinburgh, Royal Victoria HospitalEdinburgh, UK; 18 School of Social and Community Medicine and Medical Research Council and Medical Research Council Centre for Causal Analyses in Translational Epidemiology, University of BristolBristol, UK; 19 Department of Developmental and Educational Psychology, University of ValenciaValencia, Spain; 20 Department of Neuropaediatrics, La Fe University HospitalValencia, Spain; 21 University Clinic for Child and Adolescent PsychiatryEssen, Germany; 22 Department of Experimental Clinical and Health Psychology, Ghent UniversityGhent, Belgium; 23 Child and Adolescent Psychiatry, University of GöttingenGöttingen, Germany; 24 Department of Clinical Neuropsychology, Vrije UniversiteitAmsterdam, the Netherlands; 25 Institute for Disorder on Impulse and Attention, School of Psychology, University of SouthamptonSouthampton, UK; 26 Department of Child and Adolescent Psychiatry, University of ZurichZurich, Switzerland; 27 Child and Adolescent Clinical Psychology, Institute of Psychology, University of BaselBasel, Switzerland; 28 Child and Adolescent Psychiatry, Region Nordjylland Aalborg Psychiatric Hospital Aarhus, University Hospital AalborgAalborg, Denmark; 29 Department of Neuroscience, SUNY Upstate Medical UniversitySyracuse, New York; 30 Department of Psychiatry, SUNY Upstate Medical UniversitySyracuse, New York

**Keywords:** genetic association, cognition, candidate genes, ADHD, ALSPAC

## Abstract

Intelligence is a highly heritable trait for which it has proven difficult to identify the actual genes. In the past decade, five whole-genome linkage scans have suggested genomic regions important to human intelligence; however, so far none of the responsible genes or variants in those regions have been identified. Apart from these regions, a handful of candidate genes have been identified, although most of these are in need of replication. The recent growth in publicly available data sets that contain both whole genome association data and a wealth of phenotypic data, serves as an excellent resource for fine mapping and candidate gene replication. We used the publicly available data of 947 families participating in the International Multi-Centre ADHD Genetics (IMAGE) study to conduct an in silico fine mapping study of previously associated genomic locations, and to attempt replication of previously reported candidate genes for intelligence. Although this sample was ascertained for attention deficit/hyperactivity disorder (ADHD), intelligence quotient (IQ) scores were distributed normally. We tested 667 single nucleotide polymorphisms (SNPs) within 15 previously reported candidate genes for intelligence and 29451 SNPs in five genomic loci previously identified through whole genome linkage and association analyses. Significant SNPs were tested in four independent samples (4,357 subjects), one ascertained for ADHD, and three population-based samples. Associations between intelligence and SNPs in the *ATXN1* and *TRIM31* genes and in three genomic locations showed replicated association, but only in the samples ascertained for ADHD, suggesting that these genetic variants become particularly relevant to IQ on the background of a psychiatric disorder. © 2010 Wiley-Liss, Inc.

## INTRODUCTION

Intelligence is a highly heritable complex trait, for which it is hypothesized that many genes of small effect size contribute to its variability [McClearn et al., 1997; Plomin, 1999]. Almost a decade after the completion of a rough draft of the human genome sequence, major efforts have been undertaken to identify common variations related to inter-individual differences in intelligence. Plomin and coworkers [Plomin, 1999; Plomin et al., 2001, 2004; Butcher et al., 2005, 2008] conducted several genome wide association (GWA) studies and showed significant association of a functional polymorphism in *ALDH5A1* (aldehyde dehydrogenase 5 family) (MIM: 610045) on chromosome 6p with intelligence. Whole genome linkage scans for intelligence [Posthuma et al., 2005; Buyske et al., 2006; Dick et al., 2006; Luciano et al., 2006] reported two areas of genome-wide significant linkage for general intelligence on the long arm of chromosome 2 (2q24.1-31.1) and the short arm of chromosome 6 (6p25-21.2), and several areas of suggestive linkage (4p, 7q, 14q, 20p, 21p), following Lander and Kruglyak guidelines [1995]. The region on chromosome 6 (6p25-21.2) overlaps with the locus (6p24.1) identified in the genome-wide association study performed by Butcher et al. [2008]. Converging evidence from these whole genome studies provides support for the involvement of six different chromosomal regions, 2q24.1-31.1, 2q31.3, 6p25-21.2, 7q32.1, 14q11.2-12, and 16p13.3, in human intelligence (see Table I).

**TABLE I:** Summary of Genomic Loci Previously Associated With Intelligence

Locus	Refs.	Previous population
2q24.1-31.1	Posthuma et al. [2005]	Study = 1, population = 1 and 2, N = 950
	Luciano et al. [2006]	Study = 1, population = 1 and 2, N = 836
2q31.3	Butcher et al. [2008]	Study = 1, population = 3, N = 3,195
6p25-21.2	Posthuma et al. [2005]	Study = 1, population = 1 and 2, N = 950
	Luciano et al. [2006]	Study = 1, population = 1 and 2, N = 836
7q32.1	Butcher et al. [2008]	Study = 1, population = 3, N = 3,195
14q11.2-12	Buyske et al. [2006]	Study = 2, population = 1, N = 1,115
16p13.3	Butcher et al. [2008]	Study = 1, population = 3, N = 3,195

Study 1 is a family study, 2 is the COGA (Collaborative Studies on Genetics of Alcoholism) family study. Population 1 is from the Netherlands, 2 is from Australia, 3 is from the United Kingdom.

N indicates sample size.

Apart from whole genome searches, several candidate gene-based association analyses have also reported significant associations with human intelligence [for a review see Posthuma and de Geus, 2006]. Based on a literature search, we identified 16 genes that have been associated with intelligence, as measured with an intelligence quotient test (IQ) at least once (*P*-value ≤0.05); *DTNBP1* (dystrobrevin-binding protein 1) (MIM: 607145), *ALDH5A1* (aldehyde dehydrogenase 5 family, member A1) (MIM: 610045), *IGF2R* (insulin-like growth factor 2 receptor) (MIM: 147280), *CHRM2* (cholinergic muscarinic receptor 2) (MIM: 118493), *BDNF* (brain-derived neurotrophic factor) (MIM: 113505), *CTSD* (cathepsin D) (MIM: 116840), *DRD2* (dopamine receptor D2) (MIM: 126450), *KL* (klotho) (MIM: 604824), *APOE* (apolipoprotein E) (MIM: 107741), *SNAP25* (synaptosomal-associated protein, 25 kDa) (MIM: 600322), *PRNP* (prion protein (p27-30)) (MIM: 176640), *CBS* (cystathionine-beta-synthase) (MIM: 236200), *COMT* (catechol-*O*-methyltransferase) (MIM: 116790), *DNAJC13* (DnaJ (Hsp40)) (GeneID: 23317), *FADS3* (fatty acid desaturase 3) (MIM: 606150), and *TBC1D7* (TBC1 domain family, member 7) (GeneID: 51256) (see Table II).

**TABLE II:** Overview of Genes Previously Associated With Intelligence at Least Once

Gene	Chr	Gene size	SNP	Position	Type	Previous *P*-value	Refs.	Previous population
*DNAJC13*	3	*121371*	*rs1378810*	*133736780*	*Intron*	0.0007	Butcher et al. [2008]	*Study=3, population=5, N=3,195*
*TBC1D7*	6	35001	rs2496143	13419830	Intron	0.037	Butcher et al. [2008]	Study = 3, population = 5, N = 3,195
*DTNBP1*	6	140233	rs1018381	15765048	Intron	0.008	Burdick et al. [2006a, b]	Study = 1, population = 1, N = 339
*ALDH5A1*	6	42238	rs2760118	24611568	Coding-non-synonymous	0.001	Plomin et al. [2004]	Study = 4, population = 1, N = 594
*IGF2R*	6	137452	rs3832385	160446894	mrna-utr	0.02	Chorney et al. [1998]	Study = 4, population = 1, N = 102
*CHRM2*	*7*	*148372*	*rs8191992*	*136351847*	*mrna-utr*	<0.017	Comings et al. [2003]	*Study=4, population=1, N=828*
			rs8191992	136351847	mrna-utr	0.036	Dick et al. [2007]	Study = 2, population = 1, N = 1,113
			rs1378650	136355690	—	0.028	Dick et al. [2007]	Study = 2, population = 1, N = 1113
			rs1424548	136360299	—	0.037	Dick et al. [2007]	Study = 2, population = 1, N = 1113
			rs2350780	136243508	Intron	0.016	Dick et al. [2007]	Study = 2, population = 1, N = 1113
			rs2350786	136327109	Intron	0.016	Dick et al. [2007]	Study = 2, population = 1, N = 1113
			rs6948054	136331340	Intron	0.04	Dick et al. [2007]	Study = 2, population = 1, N = 1113
			rs7799047	136322097	Intron	0.02	Dick et al. [2007]	Study = 2, population = 1, N = 1113
			rs324640	136339535	Intron	<0.001	Gosso et al. [2006b]	Study = 3, population = 2, N = 667
			rs324650	136344200	Intron	<0.01	Gosso et al. [2006b]	Study = 3, population = 2, N = 667
			rs2061174	136311939	Intron	<0.01	Gosso et al. [2007]	Study = 3, population = 2, N = 762
			rs2061174	136311939	Intron	0.016	Dick et al. [2007]	Study = 2, population = 1, N = 1113
*BDNF*	11	66856	rs6265	27636491	Coding-non-synonymous	0.046	Tsai et al. [2004]	Study = 4, population = 3, N = 114
			rs6265	27636491	Coding-non-synonymous	0.001	Harris et al. [2006]	Study = 4, population = 4, N = 904
*CTSD*	11	11237	rs17571	1739169	Coding-non-synonymous	0.01	Payton et al. [2006]	Study = 4, population = 1, N = 767
*FADS3*	11	91903	rs174455	61412693	Intron	0.013	Butcher et al. [2008]	Study = 3, population = 5, N = 3195
*DRD2*	11	65564	rs2075654	112794275	Intron	0.05	Gosso et al. [2008a]	Study = 3, population = 2, N = 762
*KL*	13	49708	rs9536314	32526137	Coding-non-synonymous	0.011	Deary et al. [2005]	Study = 4, population = 4, N = 915
*APOE*	19	3611	rs28931577	50103741	Coding-non-synonymous	0.009	Deary et al. [2002]	Study = 4, population = 4, N = 466
			rs769455	50103879	Coding-non-synonymous	0.009	Deary et al. [2002]	Study = 4, population = 4, N = 466
*SNAP25*	20	88588	rs362602	10241527	—	0.005	Gosso et al. [2006a]	Study = 3, population = 2, N = 762
			rs363039	10168495	Intron	0.001	Gosso et al. [2006a]	Study = 3, population = 2, N = 762
			rs363050	10182256	Intron	0.0002	Gosso et al. [2006a]	Study = 3, population = 2, N = 762
*PRNP*	20	15437	rs1799990	4628250	Coding-non-synonymous	0.006	Kachiwala et al. [2005]	Study = 4, population = 4, N = 915
*CBS*	21	23120	rs5742905	43356252	Coding-non-synonymous	0.02	Barbaux et al. [2000]	Study = 4, Population = 1, N = 202
*COMT*	22	27221	rs4680	18331270	Coding-non-synonymous	0.05	Gosso et al. [2008a]	Study = 3, population = 2, N = 762

Study 1 is a control and case schizophrenia study, 2 is the COGA (Collaborative Studies on Genetics of Alcoholism) family study, 3 is a family study, 4 is a general population study. Population 1 is from the USA, 2 is from the Netherlands, 3 is from China, 4 is from Scotland, 5 is from the UK.

N indicates sample size.

One of the major hurdles in identifying genes for complex traits is the need for replication to distinguish false positives from genuine associations. Of all reported genetic association studies in the literature, only 4% have shown replicable association according to a 2002 search [Hirschhorn et al., 2002]. At present, searching for “genetic” and “association” in PubMed gives 69950 hits (June 2010), while adding the keywords “replicated” or “validated” results in 1,318 studies. In other words, in this rough scan around 2.0% of the total reported genetic associations are reports of validated genetic association. The field of intelligence shows no exception. Of the 16 genes mentioned above, only three (*CHRM2* [Comings et al., 2003; Gosso et al., 2006b, 2007; Dick et al., 2007], *SNAP25* [Gosso et al., 2006a, 2008b], and *BDNF* [Tsai et al., 2004; Harris et al., 2006]) have shown replicated association with intelligence across independent samples. Several other genes (e.g., *COMT*, *DTNBP1*) have repeatedly shown association to a range of cognitive traits, but have not been replicated for association with intelligence as measured with an IQ test [Small et al., 2004; Savitz et al., 2006]. The reasons for lack of replication are many and include different ethnicity, insufficient sample size, different phenotype, opposite effect direction, or the fact that no replication was attempted at all.

The recent growth in publicly available data sets that contain whole genome association data as well as a wealth of phenotypic data serves as an excellent resource for rapid replication efforts. In the public domain, the Genetic Association Information Network (GAIN)—International Multi-Centre ADHD Genetics (IMAGE) sample is the sole GWA sample with information on IQ scores. In the current article, we use data from the IMAGE project, to (a) attempt replication of previous association findings for the 16 genes associated with normal intelligence at least once, and (b) explore the six chromosome regions previously implicated in human intelligence. Associations found in the IMAGE sample (discovery sample) are subsequently attempted for replication in four independent samples. Of these four samples one is ascertained for attention deficit/hyperactivity disorder (ADHD)—as is the IMAGE sample—and three are population-based samples. This allows to investigate whether associated single nucleotide polymorphisms (SNPs) found with the IMAGE sample are discovered due to an association with intelligence in an ADHD population, or are more generally associated with intelligence.

## MATERIALS AND METHODS

### Primary Sample

Subjects of the IMAGE project have been described in detail elsewhere [Brookes et al., 2006; Kuntsi et al., 2006; Neale et al., 2008]. Briefly, 947 European Caucasian nuclear families (2,844 individuals) from eight countries (Belgium, England, Germany, Holland, Ireland, Israel, Spain, and Switzerland) were included in the analysis. Families had been recruited based on having one child with ADHD and another who would provide DNA and quantitative trait data. In addition, both parents had to be available for DNA sampling.

IQ scores were available for 606 unrelated probands (for which we also had genotyping data, see below), of which 554 were males, with a mean age of 10.99 (SD 2.74). IQ was measured with the WISC-III-R (Wechsler Intelligence Scales for children) [Wechsler, 1991] or the WAIS-III-R (Wechsler Adult Intelligence Scale) [Wechsler, 1997] when appropriate (for children aged 17 and older).

**Fig. 1:**
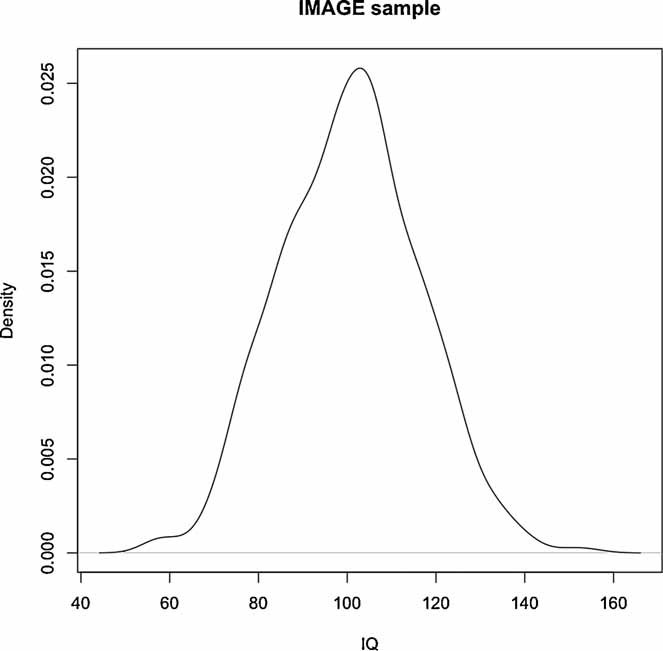
Density plot for IQ scores in the IMAGE sample.

The Verbal subtests Vocabulary and Similarities, and the Performance subtests Picture Completion and Block Design from the WISC were used to obtain an estimate of a child's IQ (prorated following procedures described by Sattler [1992]). Age-appropriate national population norms were available for each participating site included in the IMAGE sample and these were used to derive standardized estimates of intelligence [Sonuga-Barke et al., 2008]. Standardized Full-Scale IQ (FSIQ) scores had a median of 101.6 and a mean of 100.7 (SD 15.7). Skewness of the distribution of IQ scores was 0.063 while kurtosis was −0.075. The Shapiro–Wilk test was non-significant (*P* = 0.517) suggesting that the distribution of IQ in the IMAGE sample did not deviate form a normal distribution (see Fig. 1).

The parents of the probands filled out the Conner's questionnaire, which provides a quantitative measure of ADHD symptoms. Correlations between the symptom scores on the Conner's Questionnaire and IQ were −0.066 (*P* = 0.074.) for the total score, −0.029 (*P* = 0.442), for the inattention score, and −0.084 (*P* = 0.024) for the hyperactivity/impulsivity score. Although this sample was originally ascertained for ADHD, and ADHD and IQ have been reported to be associated [Frazier et al., 2004], these findings suggest that in this sample IQ scores are normally distributed (as would be expected in a population-based sample) and are at most very weakly related to ADHD symptom scores. As there were mean fluctuations across collection sites, we calculated *Z*-scores within each site/country. The use of *Z*-scores ensures that there are no mean IQ differences left across subpopulations in the IMAGE sample and therefore rules out spurious associations due to the known subpopulation structure.

### Genotyping—Primary Sample

The IMAGE study was genotyped as part of the GAIN initiative, a public–private partnership of the FNIH (Foundation for the National Institutes of Health, Inc.) that currently involves NIH, Pfizer, Affymetrix, Perlegen Sciences, Abbott, and the Eli and the Edythe Broad Institute of MIT and Harvard University (http://www.fnih.org). Genotyping was conducted at Perlegen Sciences using their genotyping platform, which comprises approximately 600,000 tagging SNPs designed to be in high linkage disequilibrium with untyped SNPs for the HapMap populations. Genotype data were cleaned by NCBI (The National Center for Biotechnology Information). Quality control analyses were processed using the GAIN QA/QC Software Package (version 0.7.4) developed by Gonçalo Abecasis and Shyam Gopalakrishnan at the University of Michigan. Details of the genotyping and data cleaning process for the IMAGE study (study accession phs000016.v1.p1) have been reported elsewhere [Neale et al., 2008].

Briefly, we selected only SNPs with a minor allele frequency (MAF) ≥0.05 and Hardy–Weinberg equilibrium (HWE) (*P* ≥ 1 × 10^−6^). Genotypes causing Mendelian inconsistencies were identified by PLINK and removed (http://pngu.mgh.harvard.edu/purcell/plink/) [Purcell et al., 2007]. We additionally removed SNPs that failed the quality control metrics for the other two GAIN Perlegen studies (i.e., Major Depression Disorder [dbGAP study accession, phs000020.v1.p1) and Psoriasis (dbGAP study accession, phs000019.v1.p1), see Neale et al., 2008]. With this filtering, 384,401 SNPs were retained in the final data set. One genomic intelligence locus (7q32.1) could not be included in the analysis because all 10 SNPs inside this relatively small area failed the quality control. The *APOE* was also not included as no SNPs were genotyped in or near this gene. Fifteen genes (*ALDH5A1*, *BDNF*, *CBS*, *CHRM2*, *COMT*, *CTSD*, *DNAJC13*, *DRD2*, *DTNBP1*, *FADS3*, *IGF2R*, *KLOTHO*, *PRNP*, *SNAP25*, and *TBC1D7*) and five genomic areas (2q24.1-31.1, 2q31.3, 6p25-21.2, 14q11.2-12, and 6p13.3) were thus included in the association analysis. From the cleaned data set, we selected all genotyped SNPs that lie in these candidate genes and genomic loci including 10 kb both upstream and downstream of each gene or genomic locus.

### Imputation

To increase coverage in the targeted genomic areas, we used the imputation approach implemented in MACH [Li et al., 2006], which imputes genotypes of SNPs that are not directly genotyped in the data set, but that are present on a reference panel. MACH is a Markov Chain-based haplotyper, which obtains an imputation of each unknown genotype using short stretches of DNA that are shared among unrelated individuals. The reference panel used was HapMap III phased data in MACH input format, which is publicly available for download from the MACH website (http://www.sph.umich.edu/csg/abecasis/MaCH/download/).

Genomic coverage of the candidate regions was extended to ∼1.5 Mb common SNPs by imputation using the HapMap phase III CEU data (NCBI build 36 (UCSC hg18)) as the reference sample. Imputed SNPs were selected if r^2^ was above 0.3 with the reference allele. Additionally, a quality threshold of 0.90 for imputation was set to be included in further association testing.

Gene coverage was determined by the sum of the typed and imputed SNPs as well as the tagged SNPs (based on HapMap information) divided by the total known common SNPs (again based on HapMap information) within a gene, using WGAviewer [Ge et al., 2008]. On average, after imputation, gene coverage was 85% in the candidate genes, with 100% coverage for *DNAJC13*, *TBC1D7*, *DTNBP1*, *ALDH5A1*, *BDNF*, and *CTSD*. In total, we analyzed 672 SNPs in the candidate genes and 29451 SNPs in the genomic loci.

### Genetic Association Analysis in IMAGE

We carried out association testing using an additive linear regression model implemented in PLINK for genotyped markers, and in MACH2QTL [Li et al., 2009], for imputed SNPs, taking into account dosage information. All IQ scores were precorrected for sex and age and no other covariates were included in the model. As mentioned above, *Z*-scores were calculated within each of the different sites included in IMAGE, such that there were no mean differences in IQ between sites. Analyses included only SNPs with a minimum 80% genotyping rate and individuals with <20% of missing genotype data. SNPs in candidate genes that had a nominal *P*-value <0.05, and the top five SNPs from the genomic regions, were selected for testing in the four replication samples.

### Replication Samples

Four replication samples totaling 4,357 independent subjects were available for replication of top findings of the IMAGE sample. One sample was ascertained for ADHD, and three samples were population-based samples.

#### DUKE cohort

The DUKE cohort consisted of 216 Americans from 108 families with a DSM-IV diagnosed ADHD-affected proband [Kollins et al., 2008]. Families were enrolled from two collection sites: Duke University Medical Center, Durham, NC, and University of North Carolina, Greensboro, NC. All participating family members provided written informed consent that had been approved by the institutional review board at the ascertaining institution. The WAIS-III was administered to individuals 17 years of age or older, and the WISC-IV was given to children ages 6–16. The Wechsler Preschool and Primary Scale of Intelligence—3rd edition (WPPSI-III) was used for children under the age of 6 [Wechsler, 2002]. FSIQ was estimated for both adults and children from the vocabulary and block design subtests (M = 109.5 and SD = 12.9). Parents and children were genotyped using the Illumina Infinium HumanHap300 duo chip (Illumina, Inc., San Diego, CA). Quality of the Illumina data was assessed using PLINK (http://pngu.mgh.harvard.edu/purcell/plink/) [Purcell et al. 2007]. SNPs (315,980) were submitted for quality checks. Call rates exceeded 98% for all individuals, one individual was excluded due to a gender discrepancy, and two individuals were excluded due to per-family Mendelian errors in excess of 1%. Out of the 315,980 SNPs submitted, 6,109 SNPs were excluded based on a MAF <0.05, 13 SNPs were excluded due to Mendelian errors in >4 families, and 629 SNPs were excluded due to deviations from HWE (*P* < 0.000001). In total, 3 individuals and 6,751 SNPs did not pass our quality control checks. Two Centre d'Etude du Polymorphism Humain (CEPH) controls and blinded duplicates were used for every 94 samples and required to match 100%. Data were genome-wide imputed with the use of the phased data from the HapMap samples (CEU; build 36, release 22) and MACH. Association analysis was carried out using QTDT (http://www.sph.umich.edu/csg/abecasis/QTDT/). QTDT adopts the between/within model as used by Fulker et al. [1999] and Purcell et al. [2007] as implemented in the QFAM package. We tested for population stratification by comparing the between and within family components of association, using a variant of the orthogonal model [Abecasis et al., 2000]. None of the tested SNPs showed sign of stratification in this population.

#### ALSPAC sample

The Avon Longitudinal Study of Parents and Children (ALSPAC) is a large population-based, prospective birth cohort consisting initially of over 13,000 women and their children recruited from the Bristol area, UK in the early 1990s [Golding et al., 2001]. ALSPAC has extensive data collections on health and development of children and their parents from the 8th gestational week onwards. Ethical approval for the study was obtained from the ALSPAC Law and Ethics Committee and the local research ethics committees. FSIQ within ALSPAC was measured at the age of 8 with the WISC-III [Wechsler et al., 1992]. A short version of the test consisting of alternate items only (except the coding task) was applied by trained psychologists [Joinson et al., 2007]. Verbal (Information, Similarities, Arithmetic, Vocabulary, and Comprehension) and Performance (Picture Completion, Coding, Picture arrangement, Block Design, and Object assembly) subtests were administered; the subtests were scaled and scores for FSIQ derived. ALSPAC (1,543) children were initially genotyped at 317,504 SNPs on the Illumina HumanHap317K SNP chip. Individuals exhibiting cryptic relatedness, non-European ancestry, high genome-wide heterozygosity, and/or missing rates were excluded as described in Timpson et al. [2009], leaving 1,518 individuals in the analysis of whom 1,495 had information on FSIQ within a range of ±4 SD (M = 106.8, SD = 15.6). Markers with MAF <1%, SNPs with >5% missing genotypes and markers that failed an exact test of HWE (*P* < 5 × 10^−6^) were excluded from further analyses leaving 310,505 SNPs that passed quality control. GWAS analysis was performed on sex and population stratification-adjusted (first five principal components from Eigenstrat analysis) [Price et al., 2006] *Z*-standardized IQ scores. Genome-wide imputation was done using the HapMap phase I-II CEU data (release 22, NCBI build 36) as the reference sample and MACH software.

#### QIMR adolescent (Brisbane Adolescent Twin and Family) sample

The QIMR adolescent cohort is a population-based cohort, consisting of 1,670 Australians (793 male, 877 female) from 741 families with mean age of 16.4 (SD = 4). FSIQ was assessed with the Multidimensional Aptitude Battery [MAB; Jackson, 1984]. Five subtests were administered (three Verbal: Information, Arithmetic, Vocabulary; two Performance: Spatial, Object Assembly) and from these a standardized FSIQ measure was obtained. FSIQ had a mean of 112.6 (SD = 12.8). Genotyping was done using the Illumina 610K SNP platform and Illumina BeadStudio software, with 529,721 SNPs passing QC. Data were imputed to ∼2.3 million SNPs with the use of the phased data from the HapMap samples (CEU; build 36, release 22) and MACH.9, described in detail in Medland et al. [2009], (see Project 5: ADOL deCODE). Individual SNPs were tested for association with the family-based score test implemented in Merlin. This study was approved by the QIMR human research ethics committee and informed written consent was obtained from all participants.

#### Lothian Birth Cohort 1936 (LBC1936) sample

The LBC1936 consisted of 1,091 individuals who, at the age of ∼11 years, participated in the Scottish Mental Survey of 1947, when they took a validated mental ability test, the Moray House Test No. 12 (MHT). Briefly, at a mean age of 69.6 years (SD = 0.8) participants of LBC1936 were recruited to a study to investigate the causes of cognitive ageing. They underwent a series of cognitive, physical, and biochemical tests at the Wellcome Trust Clinical Research Facility (WTCRF) at the Western General Hospital, Edinburgh. For this study, a general cognitive ability factor was derived from principal components analysis of six Wechsler Adult Intelligence Scale-III^UK^ (WAIS-III) subtests (matrix reasoning, letter number sequencing, block design, symbol search, digit span backwards, and digit symbol), as described previously [Luciano et al., 2009]. The general cognitive ability factor scores were corrected for age in days and sex, and converted to IQ scores (mean = 100; SD = 15). DNA was isolated by standard procedure at the WTCRF Genetics Core, Western General Hospital, Edinburgh from 1,071 individuals. Twenty-nine samples failed quality control preceding the genotyping procedure. The remaining 1,042 samples (all blood-extracted) were genotyped at the WTCRF Genetics Core using the Illumina610-Quadv1 chip. These samples were then subjected to the following quality control procedures after which 1,005 samples remained. All individuals were checked for disagreement between genetic and reported gender (n = 12). Relatedness between subjects was investigated and, for any related pair of individuals, one was removed (n = 8). Samples with a call rate ≤0.95 (n = 16), and those showing evidence of non-Caucasian ascent by multidimensional scaling, were also removed (n = 1). SNPs were included in the analyses if they met the following conditions: call rate ≥0.98, MAF ≥0.01, and HWE test with *P* ≥ 0.001. The final number of SNPs included in the genome-wide association study was 549,091. IQ scores and genotype were available for 976 individuals. Genomic coverage was extended to ∼2.5 million common SNPs by imputation using the HapMap phase II CEU data (NCBI build 36 (UCSC hg18)) as the reference sample and MACH software. SNPs with low imputation (r^2^ < 0.30), low MAF (<0.01), and divergence from HWE (*P* < 0.001) were excluded so that respective SNP and sample call rates were 0.98 and 0.95.

#### Statistical power

The primary (IMAGE) sample of 606 subjects had sufficient (80%) statistical power to detect SNPs that explained at least 1.3% of the variance for direct replication (significance level 0.05) (Genetic Power Calculator) [Purcell et al., 2003], which is in the order of effect sizes of SNPs reported previously. The sample size of the meta-analysis including the two ADHD samples (606 + 216 = 822) was sufficient to detect genetic effects explaining 2% of the variance, given a Bonferroni corrected significance level of 0.001. The sample size including all samples (N = 4,963) was sufficient to detect SNPs explaining 0.35% (i.e., <1%) of the variance (significance level of 0.001).

#### Replication analysis

All populations were imputed using MACH and imputed SNPs were included in our analysis if quality score > 0.9 and r^2^ > 0.3 and MAF > 0.05. IQ scores were all corrected for effects of age and sex and transformed to *Z*-scores and standardized such that the mean was 100 and SD = 15, within each sample, for comparison of effect sizes across samples.

#### Meta-analysis

Although replication across different samples provides information on the genuineness of an initial association, meta-analysis appropriately weighs the effect and sample sizes across different replications samples. We thus conducted a meta-analysis, in which the primary sample was included to increase statistical power [Skol et al., 2006]. We used a stepwise approach, in which we first ran a combined analysis based on the two samples ascertained for ADHD, and then conducted a meta-analysis on all 4,963 subjects. The meta-analysis was conducted using the METAL program (http://www.sph.umich.edu/csg/abecasis/metal/). METAL creates a single summary *P*-value for each SNP from all samples together. For each marker, an arbitrary reference allele is selected and a *Z*-statistic, characterizing the evidence for association, is used as input. The *Z*-statistic summarizes the magnitude and the direction of an effect relative to the reference allele. An overall *Z*-statistic and *P*-value are then calculated from the weighted average of the individual statistics. Weights are proportional to the square root of the number of individuals examined in each sample, and selected such that the squared weights sum to 1.0. Outcomes of the meta-analyses were tested against a Bonferroni corrected threshold of significance (*P* < 0.001).

## RESULTS

### Primary Sample—IMAGE Cohort

Most previously reported associations of genes with intelligence included intronic SNPs with no clear function. This suggests that they might be controlling RNA signaling networks or that other SNPs in LD might be the actual causal variant. We used imputation to increase coverage. We do note; however, that even after imputation, not all of the originally reported SNPs were available in the current sample. Of the 15 candidate genes, six genes showed at least one SNP with a *P*-value <0.05 (see Table III).

**TABLE III:** Results of 15 Candidate Genes for Intelligence in the IMAGE Cohort

GENE	Previous associated SNP (G/I)^a^	*P*-value with previous associated SNP	nSNPs tested	Coverage	SNP density, kb/SNP	nSNPs, *P* < 0.05	Most significant SNP (G/I)^a^	Position	Type	Best *P*-value
*DNAJC13*	rs1378810 (I)	0.642	45	1	31.41	0	rs12637073 (I)	133666251	Intronic	0.096
*TBC1D7*	rs2496143 (I)	0.8568	47	1	9.20	0	rs480122 (G)	13425063	Intronic	0.588
*DTNBP1*	rs1018381	—	65	1	24.55	5	rs760666 (G)	15589121	Intronic	0.020
*ALDH5A1*	rs2760118 (I)	0.8328	46	1	13.52	1	rs2760138 (I)	24620816	Intronic	0.047
*IGF2R*	rs3832385	—	88	0.967	18.62	5	rs8191898 (I)	160418955	Intronic	0.018
*CHRM2*	rs8191992	—	81	0.942	21.10	2	rs6467694 (G)	136197456	Upstream	0.010
	rs1378650 (G)	0.8284								
	rs1424548 (I)	0.3888								
	rs2350780 (I)	0.9788								
	rs2350786 (G)	0.6316								
	rs6948054 (I)	0.322								
	rs7799047	—								
	rs324640	—								
	rs324650 (I)	0.2514								
	rs2061174	—								
*BDNF*	rs6265 (G)	0.1018	29	1	29.86	2	rs12288512 (I)	27704247	Upstream	0.011
*CTSD*	rs17571 (G)	0.2932	6	1	59.01	0	rs3740621 (I)	1728373	Upstream	0.081
*FADS3*	rs174455 (I)	0.6665	9	0.875	41.54	0	rs174626 (G)	61393633	Downstream	0.050
*DRD2*	rs2075654	—	57	0.982	15.02	2	rs4630328 (I)	112839419	Intronic	0.047
*KL*	rs9536314 (G)	0.6873	52	0.933	13.39	0	rs17763040 (G)	32543384	Intergenic	0.142
*SNAP25*	rs362602 (G)	0.2254	71	0.913		0	rs362990 (G)	10224221	Intronic	0.063
	rs363039 (I)	0.6062								
	rs363050 (G)	0.3723								
*PRNP*	rs1799990 (G)	0.944	21	0.778	20.63	0	rs6084833 (I)	4620759	Intronic	0.135
*CBS*	rs5742905	—	22	0.675	19.83	0	rs1788490 (I)	43340620	Intergenic	0.189
*COMT*	rs4680 (G)	0.6209	33	0.633	14.62	0	rs9332377 (I)	18335692	Intronic	0.08

Genome build 36.

a G and I indicate genotyped and imputed SNPs, respectively.

Of the five previously reported genomic loci (2q24.1-31.1, 2q31.3, 6p25-21.2, 14q11.2-12, and 16p13) investigated here, we observed *P*-values <0.0025 in three regions (6p25-21.2, 2q24.1-31.1, and 14q11.2-12) (see Table IV). Genomic areas 2q31.3 and 16p13.3 showed no association with IQ (all *P*-values >0.15). On a SNP level, there were three independent SNPs in intergenic and non-coding regions with *P*-values ≤2.0 × 10^−4^ inside the 2q24.1-31.1 and 14q11.2-12 areas (Table IV). The lowest *P*-values were observed for rs2807822, *P* = 1 × 10^−4^; rs4972741, *P* = 1.7 × 10^−4^; and rs6721348 *P* = 1.8 × 10^−4^.

**TABLE IV:** Replication Results in the Genomic Loci Previously Associated With Intelligence in the IMAGE Cohort

SNP (G/I)^a^	Minor/major allele	MAF	Rank	P-value	Chr	Position	Type	Closest gene	Distance to gene
Genomic location 2q24.1-31.1 (from 154475832 to 177730691 bp) total SNPs tested = 7,819 in 182 genes									
rs4972741 (I)	G/A	0.12	1	0.00017	2	172823906	Intergenic	*AC104088.1*	−64,355
rs6721348 (I)	C/T	0.12	2	0.00018	2	172826755	Intergenic	*AC104088.1*	−61,506
rs10172929 (G)	G/T	0.13	3	0.00031	2	164756952	Intergenic	*AC092684.1*	0
rs16844374 (G)	C/T	0.15	7	0.00127	2	160394348	Intronic	*LY75*	0
rs10201330 (I)	T/C	0.09	4	0.00132	2	177056271	Intergenic	*AC017048.3*	22,948
rs4289149 (G)	A/G	0.18	8	0.00150	2	172834736	Intergenic	*ITGA6*	165,264
rs995711 (G)	G/T	0.12	5	0.00174	2	164123635	Intergenic	*FIGN*	−34,517
rs11896469 (G)	C/T	0.44	6	0.00230	2	176388492	Intergenic	*EXTL2P1*	27,369
Genomic location 6p25-21.2 (from 5945435 to 41007859 bp) total SNPs tested = 18,651 in 809 genes									
rs12204969 (I)	C/T	0.12	1	0.00018	6	16802156	Intronic	*ATXN1*	0
rs17606216 (G)	C/T	0.12	2	0.00018	6	16796594	Intronic	*ATXN1*	0
rs993600 (G)	G/A	0.16	3	0.00027	6	22153623	Within non-coding gene	*RP1-67M12.1*	0
rs2023472 (G)	A/G	0.42	4	0.00028	6	30183843	Intergenic	*TRIM31*	5,241
rs6929819 (G)	G/A	0.43	5	0.00033	6	33670832	Intergenic	*C6orf227*	1,739
rs195371 (G)	G/A	0.23	6	0.00034	6	37412364	Intergenic	*TBC1*	3,464-
rs6929774 (I)	T/C	0.42	7	0.00039	6	33670698	Intergenic	*C6orf227*	1,605
rs17606174 (G)	T/C	0.13	8	0.00050	6	16795524	Intronic	*ATXN1*	0
Genomic location 14q11.2-12 (from 21269202 to 28322992 bp) total SNPs tested = 2,964 in 233 genes									
rs2807822 (I)	T/C	0.47	1	0.00010	14	27554764	Intergenic	*AL445384.1*	25,600
rs3811222 (I)	A/G	0.10	2	0.00066	14	22020854	Intronic	*TRAC*	0
rs762578 (I)	T/G	0.11	3	0.00069	14	22020088	Intronic	*TRAC*	0
rs1872159 (G)	T/C	0.09	4	0.00080	14	22017743	Intronic	*TRAC*	0
rs7149201 (I)	C/T	0.20	5	0.00178	14	23034259	Intergenic	*NGDN*	17,017
rs877726 (G)	T/A	0.23	6	0.00230	14	27557719	Intergenic	*AL445384.1*	−28,555

Only SNPs with a *P* < 0.0025 are shown.

Genome build 36.

a G and I indicate genotyped and imputed SNPs, respectively.

To confirm whether the nominally significant SNPs (*P* < 0.05) from the candidate genes and the top SNPs (*P* < 0.0025) in each of the genomic regions with IQ were simply due to chance, we tested these SNPs in the replication samples.

### Replication of Primary Associations in Candidate Genes and Genomic Areas

We attempted replication in four independent cohorts. We first performed an association analysis of the 17 nominally associated SNPs (*P*-value <0.05) in the candidate genes, and the 22 most strongly associated SNPs in the genomic areas in each population (total of 39 SNPs), using the same reference allele for each SNP across different populations. The MAF of the tested SNPs across the five samples were comparable (see Supplementary Table S1).

We first conducted a combined analysis on only the two samples ascertained for ADHD. We then combined all five samples to test whether the significant SNPs were associated with intelligence in a general context, or merely in an ADHD background. Although IQ was normally distributed in both samples ascertained for ADHD, association of a SNP with IQ in an ADHD background may differ from association of that SNP with intelligence in a non-ADHD background.

When combining the two samples ascertained for ADHD we found that of all tested SNPs, 12 had a *P*-value <0.05 (same direction of effect) of which 6 showed evidence for associated after Bonferroni correction (*P* < 0.001) for multiple testing. For one of these SNPs (rs2807822, intergenic, 14q11.2-12), however, the effect was in opposite direction in the two samples ascertained for ADHD, also indicated by a significant heterogeneity effect (*P* = 0.04; see Supplementary Table S2). Three other SNPs were in intergenic areas 6p25-21.2 (one SNP) and 14q11.2-12 (two SNPs), while two SNPs were in genic areas: rs17606174 (*P* = 0.00018), located in the second intron of *ATXN1* (ataxin 1) (MIM: 601556), and rs2023472 (*P* = 0.0003), located in exon 5 on *TRIM31* (tripartite motif-containing 31) (MIM: 609316). Allelic effect sizes were in the order of 3–4 IQ points in the combined DUKE and IMAGE samples. When we combined all five samples, none of these associations were significant, even though some of the SNPs showed similar direction of effects in some of the replication samples. We provide results in Table V.

**TABLE V:** Meta-Analysis of the Top SNPs From the Primary Analysis

				IMAGE (N = 606)		DUKE (N = 216)		ALSPAC (N = 1,495)		QIMR (N = 1,670)		LBC1936 (N = 976)		IMAGE and DUKE (N = 822)		ALL (N = 4,963)	
										
Gene	Genomic area	SNP	A	B	*P*	B	*P*	B	*P*	B	*P*	B	*P*	*Z*	*P*	*Z*	P
Intergenic	2q24.1-31.1	rs10172929	T	4.64	0.0003			0.23	0.84	−0.03	0.96	−0.94	0.35			0.95	0.34
Intergenic	2q24.1-31.1	rs10201330	T	5.43	0.0013			1.28	0.27	−0.76	0.38	0.19	0.88			1.32	0.19
*CHRM2*	7q33	rs10271552	T	−3.18	0.0398			−0.42	0.65	−0.23	0.76	−1.38	0.23			−1.72	0.09
Intergenic	2q24.1-31.1	rs11896469	T	2.55	0.0023			−0.01	0.71	0.47	0.32	−0.38	0.58			1.18	0.24
*ATXN1*	6p25-21.2	rs12204969	T	4.98	0.0002			−2.12	0.01	−0.99	0.14	0.49	0.61			0.65	0.51
*BDNF*	11p14	rs12273363	T	−2.61	0.0131	0.88	0.68	−0.42	0.54	0.13	0.82	0.70	0.43	−2.00	0.0456	0.95	0.34
*BDNF*	11p14	rs12288512	A	2.67	0.0114	−0.88	0.68	0.42	0.55	−0.13	0.82	−0.70	0.43	2.04	0.0410	1.32	0.19
*LY75*	2q24.1-31.1	rs16844374	T	3.77	0.0013												
*ATXN1*	6p25-21.2	rs17606174	T	−4.47	0.0005	−3.95	0.16	0.12	0.02	0.40	0.53	−0.76	0.41	−3.80	**0.0001**	−0.22	0.83
*ATXN1*	6p25-21.2	rs17606216	T	4.91	0.0002			−2.15	0.01	−0.86	0.20	0.43	0.65			−0.67	0.51
*IGF2R*	6q26	rs1805075	A	−4.47	0.0189			−0.30	0.82	0.76	0.50	−0.62	0.71			−0.73	0.47
Intergenic	14q11.2-12	rs1872159	T	4.77	0.0008	2.61	0.46	−0.30	0.76	0.58	0.43	0.75	0.47	3.32	**0.0009**	1.92	0.06
Intergenic	6p25-21.2	rs195371	A	−3.65	0.0003			−0.14	0.00	0.62	0.28	−0.23	0.78			−2.41	0.02
*TRIM31*	6p25-21.2	rs2023472	A	−3.09	0.0003	−1.58	0.35	0.19	0.75	0.95	0.05	0.23	0.73	−3.65	**0.0003**	−0.25	0.80
*DTNBP1*	6p23	rs2619545	T	−2.34	0.0362	2.07	0.34	0.25	0.73	−0.34	0.56	0.87	0.30	−1.41	0.1589	−1.98	0.05
*ALDH5A1*	6p23	rs2760138	A	−3.05	0.0478												
Intergenic	14q11.2-12	rs2807822	T	−3.63	0.0000	0.39	0.82	0.09	0.89	0.77	0.10	−0.19	0.78	−3.34	0.0009	0.54	0.59
Intergenic	14q11.2-12	rs3811222	A	5.15	0.0007	0.91	0.79	−0.47	0.68	0.59	0.41	0.98	0.33	3.16	0.0016	−1.87	0.06
Intergenic	2q24.1-31.1	rs4289149	A	3.36	0.0015	−3.52	0.14	0.00	0.99	1.18	0.05	−0.62	0.48	2.08	0.0380	1.67	0.10
*DRD2*	11q23	rs4630328	A	−1.79	0.0475			0.95	0.14	0.18	0.71	0.33	0.66			−0.74	0.46
Intergenic	2q24.1-31.1	rs4972741	A	−5.51	0.0002			−1.15	0.24	−0.23	0.78	0.80	0.51			−1.87	0.06
*CHRM2*	7q33	rs6467694	T	−3.86	0.0097	−1.47	0.59	0.92	0.32	1.11	0.14	−0.94	0.41	−2.54	0.0112	−0.73	0.47
*DRD2*	11q23	rs6589377	A	1.71	0.0504	−0.29	0.87	−0.92	0.13	−0.18	0.71	−0.53	0.46	1.65	0.0986	1.92	0.06
Intergenic	2q24.1-31.1	rs6721348	A	−5.51	0.0002			−1.09	0.26	−0.28	0.73	0.79	0.51			0.04	0.97
Intergenic	6p25-21.2	rs6929774	T	−3.09	0.0004	1.98	0.23	0.05	0.18	−0.63	0.19	0.43	0.53	−2.62	0.0089	−0.77	0.44
*C6orf227*	6p25-21.2	rs6929819	A	3.08	0.0003	−1.09	0.26	−0.05	0.20	0.65	0.18	−0.41	0.55	2.65	0.0081	0.86	0.39
Intergenic	14q11.2-12	rs7149201	T	3.72	0.0018	−2.30	0.42	0.36	0.62	−0.39	0.49	0.49	0.54	2.45	0.0144	1.10	0.27
*DTNBP1*	6p23	rs760666	A	−2.34	0.0223	0.87	0.69	−0.33	0.64	0.13	0.81	−0.11	0.90	−1.85	0.0644	−0.91	0.36
Intergenic	14q11.2-12	rs762578	T	4.62	0.0007	2.32	0.40	−0.31	0.73	0.73	0.29	0.27	0.79	3.40	**0.0007**	1.89	0.06
*DTNBP1*	6p23	rs7758659	T	−2.34	0.0225	0.88	0.69	−0.34	0.63	0.13	0.81	−0.11	0.89	−1.85	0.0648	−0.91	0.36
*IGF2R*	6q26	rs8191818	T	−4.49	0.0200	−2.79	0.35	−0.36	0.80	0.65	0.56	−0.47	0.78	−2.49	0.0126	−0.92	0.36
*IGF2R*	6q26	rs8191821	T	4.52	0.0196	2.79	0.35	0.34	0.80	−0.65	0.56	0.47	0.78	2.50	0.0124	0.92	0.36
*IGF2R*	6q26	rs8191898	T	4.47	0.0185	2.78	0.35	0.31	0.82	−0.76	0.50	0.47	0.78	2.52	0.0117	0.86	0.39
*DTNBP1*	6p23	rs875462	T	2.21	0.0318	−1.20	0.57	0.39	0.57	−0.17	0.76	0.59	0.47	1.64	0.1012	1.11	0.27
Intergenic	14q11.2-12	rs877726	A	−3.09	0.0023	−0.55	0.79	0.00	0.92	0.65	0.23	−0.41	0.59	−2.75	0.0059	−0.67	0.50
*DTNBP1*	6p23	rs9296983	A	−2.33	0.0229	0.87	0.69	−0.33	0.64	0.16	0.77	−0.11	0.90	−1.84	0.0657	1.10	0.27
*IGF2R*	6q26	rs9457827	T	4.47	0.0187	2.78	0.35	0.30	0.82	−0.76	0.50	0.62	0.71	2.52	0.0119	0.89	0.37
Non-coding gene	6p25-21.2	rs993600	A	4.11	0.0003	1.41	0.49	0.33	0.67	0.00	0.99	0.13	0.88	3.55	**0.0004**	1.71	0.09
Intergenic	2q24.1-31.1	rs995711	T	4.02	0.0018	2.16	0.44	1.19	0.25	−0.67	0.40	0.27	0.84	3.15	0.0017	1.47	0.14

A is the reference allele; B is the beta effect; *P* is the *P*-value, *Z* is the *Z*-statistic as provided in the meta-analysis.

Bold indicates below the Bonferroni corrected threshold of significance (<0.001) and same direction of effect in the meta-analysis.

## DISCUSSION

This study aimed to replicate association of previously reported candidate genes for IQ as well as to fine-map previously linked genomic areas. As available samples differed in ascertainment method (i.e., ascertained for ADHD or population based) we tested for SNP associations with IQ in an ADHD background and in a non-ADHD, general population, background.

In the primary analysis, we found weak evidence for the association of some of the previously reported genes with IQ: *IGF2R* (five SNPs with *P*-value <0.05), *DTNBP1* (five SNPs with *P*-value <0.05), ALDHA5A1 (one SNP with *P*-value <0.05), *BDNF* (two SNPs with *P*-value <0.05), *DRD2* (two SNPs with *P*-value <0.05), and *CHRM2* (two SNPs with *P*-value ≤0.03). None of SNPs previously associated with IQ showed association in the current study (*P*-value >0.05). The lack of replication can either indicate a false positive finding in previous studies, or might be explained by the ascertainment for ADHD in our primary sample. Although association between IQ and ADHD in the current sample was not significant, and IQ was distributed normally in the IMAGE sample, previous reports [e.g., Kuntsi et al., 2004] do indicate a (genetic) association between ADHD and IQ.

Results from the primary association analysis in the genomic loci implicated three intergenic regions (2q24.1-31, 6p25-21.2, and 14q11.2-12). The nominally significant SNPs from the candidate genes, and the top SNPs from the genomic regions, were included in a stepwise combined analysis. When we combined the two samples ascertained for ADHD (totaling 822 subjects), we found that five SNPs were associated with IQ. None of these SNPs were inside candidate genes previously implicated, but instead were located in two genomic areas: 6p25-21.2 and 14q11.2-12. Two of these SNPs were inside two genes: rs17606174 was in the second intron of the *ATXN1* gene, and rs2023472 in exon 5 on *TRIM31*. However, when we combined all samples, none of these SNPs showed a significant association with intelligence. However, we cannot exclude the possibility of type I error given the total number of tests performed within the discovery sample only. These results provide suggestive evidence that the ATXN1 and TRIM31 genes, and several other SNPs in areas 6p25-21.2 and 14q11.2-12, are related to IQ, but only on the background of ADHD.

In the primary IMAGE association results, *ATXN1* has 25 SNPs with *P*-value <0.05, and most of them are located in the second intron of *ATXN1*, nearby an alternative splicing region. ATXN1 is present in the nucleus of the neurons of the basal ganglia, pons and cortex, and in both cytoplasm and nucleus of Purkinje cells of the cerebellum [Servadio et al., 1995]. Expansion of a (CAG)n repeat in *ATXN1* (previous called *SCA1* gene) causes spinocerebellar ataxia-1 (*SCA1*) in humans (MIM: 164400) [Orr et al., 1993; Banfi et al., 1994]. It was also reported that mice lacking *ATXN1* are characterized by decreased exploratory behavior, pronounced deficits in the spatial version of the Morris water maze test, and impaired performance on the rotating rod apparatus [Matilla et al., 1998], pointing to the possible role of *ATXN1* in learning and memory.

In the primary IMAGE association results, *TRIM31* has 23 SNPs with *P*-value <0.05 and most of them are located in the 5′ region and in intron 1 of *TRIM31*. The protein encoded by this gene is a member of the tripartite motif (TRIM) family. The TRIM motif includes three zinc-binding domains, a RING, a B-box type 1 and a B-box type 2, and a coiled-coil region [Meroni and Diez-Roux, 2005]. Other members of the TRIM family (TRIM3, MIM: 605493) were reported to modulate NGF-induced neurite outgrowth in PC12 cells [El-Husseini and Vincent, 1999].

In summary, we found very little support for genetic variants in genes that have previously been associated with intelligence. In addition, this study did provide tentative support for a role of the *ATXN1* and *TRIM31* genes in previously associated linkage areas for intelligence in the context of a psychiatric disorder, that is, ADHD. This suggests that genetic variants important for IQ in a non-psychiatric population may not necessary overlap with genetic variants important for IQ in a psychiatric population.
