# Supplementary material for: The ATXN1 and TRIM31 genes are related to intelligence in an ADHD background: Evidence from a large collaborative study totaling 4,963 Subjects
Source: Am J Med Genet B Neuropsychiatr Genet. 2010 Dec 16;156(2):145–57. doi: 10.1002/ajmg.b.31149 (PMC3085124; doi:10.1002/ajmg.b.31149)
Supplement: Supplementary file 1 [file ajmg0156-0145-SD1.doc]

**Table S1.** Minor allele frequencies across samples of the SNPs included in the replication analysis.

| **GENE** | **Genomic area** | **SNP** | **MAJOR/MINOR** | **MAF_IMAGE** | **MAF_DUKE** | **MAF_ALSPAC** | **MAF_QIMR** | **MAF_LBC1936** |
| --- | --- | --- | --- | --- | --- | --- | --- | --- |
| Intergenic | 2q24.1-31.1 | rs10172929 | T/G | 0.1399 |  | 0.1354 | 0.1183 | 0.1245 |
| Intergenic | 2q24.1-31.1 | rs10201330 | C/T | 0.09 |  | 0.0974 | 0.07907 | 0.07326 |
| *CHRM2* | 7q33 | rs10271552 | T/C | 0.091 |  | 0.1062 | 0.1044 | 0.09785 |
| Intergenic | 2q24.1-31.1 | rs11896469 | T/C | 0.4472 |  | 0.44 | 0.4266 | 0.4175 |
| *ATXN1* | 6p25-21.2 | rs12204969 | T/C | 0.1217 |  | 0.1358 | 0.1303 | 0.1383 |
| *BDNF* | 11p14 | rs12273363 | T/C | 0.192 | 0.2201 | 0.2185 | 0.2026 | 0.1967 |
| *BDNF* | 11p14 | rs12288512 | G/A | 0.1962 | 0.2199 | 0.2184 | 0.2025 | 0.1967 |
| *LY75* | 2q24.1-31.1 | rs16844374 | T/C | 0.15 |  |  |  |  |
| *ATXN1* | 6p25-21.2 | rs17606174 | C/T | 0.1346 | 0.1113 | 0.15 | 0.1458 | 0.1568 |
| *ATXN1* | 6p25-21.2 | rs17606216 | T/C | 0.1229 |  | 0.1365 | 0.1297 | 0.1368 |
| *IGF2R* | 6q26 | rs1805075 | A/G | 0.0594 |  | 0.0515 | 0.04628 | 0.04508 |
| Intergenic | 14q11.2-12 | rs1872159 | C/T | 0.098 | 0.1258 | 0.1075 | 0.1135 | 0.1265 |
| Intergenic | 6p25-21.2 | rs195371 | A/G | 0.2341 |  | 0.22 | 0.2167 | 0.208 |
| *TRIM31* | 6p25-21.2 | rs2023472 | G/A | 0.4212 | 0.3889 | 0.395 | 0.4089 | 0.3934 |
| *DTNBP1* | 6p23 | rs2619545 | T/C | 0.2058 | 0.1871 | 0.2085 | 0.2017 | 0.208 |
| *ALDH5A1* | 6p23 | rs2760138 | A/G | 0.1016 |  |  |  |  |
| Intergenic | 14q11.2-12 | rs2807822 | C/T | 0.4753 | 0.4449 | 0.4701 | 0.4901 | 0.4672 |
| Intergenic | 14q11.2-12 | rs3811222 | G/A | 0.1045 | 0.1252 | 0.1071 | 0.1139 | 0.1358 |
| Intergenic | 2q24.1-31.1 | rs4289149 | G/A | 0.18 | 0.1775 | 0.19 | 0.1886 | 0.1814 |
| *DRD2* | 11q23 | rs4630328 | G/A | 0.3698 |  | 0.3538 | 0.3517 | 0.3294 |
| Intergenic | 2q24.1-31.1 | rs4972741 | A/G | 0.1216 |  | 0.0958 | 0.09398 | 0.0835 |
| *CHRM2* | 7q33 | rs6467694 | T/C | 0.0939 | 0.1202 | 0.1027 | 0.1046 | 0.0958 |
| *DRD2* | 11q23 | rs6589377 | A/G | 0.386 | 0.3643 | 0.3826 | 0.3705 | 0.3463 |
| Intergenic | 2q24.1-31.1 | rs6721348 | A/G | 0.1216 |  | 0.0971 | 0.09448 | 0.0835 |
| Intergenic | 6p25-21.2 | rs6929774 | C/T | 0.4274 | 0.4575 | 0.45 | 0.4652 | 0.4657 |
| *C6orf227* | 6p25-21.2 | rs6929819 | A/G | 0.435 | 0.4653 | 0.4673 | 0.4675 | 0.4667 |
| Intergenic | 14q11.2-12 | rs7149201 | T/C | 0.1935 | 0.1968 | 0.2229 | 0.2287 | 0.2269 |
| *DTNBP1* | 6p23 | rs760666 | G/A | 0.2471 | 0.2254 | 0.2168 | 0.2355 | 0.2136 |
| Intergenic | 14q11.2-12 | rs762578 | G/T | 0.1141 | 0.1353 | 0.1231 | 0.1289 | 0.1424 |
| *DTNBP1* | 6p23 | rs7758659 | C/T | 0.2478 | 0.2254 | 0.2169 | 0.2355 | 0.2136 |
| *IGF2R* | 6q26 | rs8191818 | T/G | 0.0537 | 0.0859 | 0.0504 | 0.04623 | 0.04508 |
| *IGF2R* | 6q26 | rs8191821 | C/T | 0.0542 | 0.086 | 0.0505 | 0.04623 | 0.04508 |
| *IGF2R* | 6q26 | rs8191898 | C/T | 0.0596 | 0.087 | 0.0514 | 0.04628 | 0.04508 |
| *DTNBP1* | 6p23 | rs875462 | T/C | 0.2518 | 0.24 | 0.2321 | 0.2353 | 0.2223 |
| Intergenic | 14q11.2-12 | rs877726 | A/T | 0.2351 | 0.263 | 0.27 | 0.257 | 0.2649 |
| *DTNBP1* | 6p23 | rs9296983 | G/A | 0.2471 | 0.2253 | 0.2168 | 0.2351 | 0.2136 |
| *IGF2R* | 6q26 | rs9457827 | C/T | 0.0595 | 0.0872 | 0.0515 | 0.04628 | 0.04508 |
| Noncoding gene | 6p25-21.2 | rs993600 | A/G | 0.1689 | 0.2204 | 0.1702 | 0.1969 | 0.1926 |
| Intergenic | 2q24.1-31.1 | rs995711 | T/G | 0.1253 | 0.1315 | 0.1174 | 0.09851 | 0.09324 |

***Table S2.*** *Heterogeneity tests in the meta-analysis.*

| **GENE** | **Genomic area** | **SNP** | **IMAGE & DUKE** | | | **ALL** | | |
| --- | --- | --- | --- | --- | --- | --- | --- | --- |
|  |  |  | **N=822** | | | **N=4963** | | |
|  |  |  | **HetChiSq** | **HetDf** | **HetPVal** | **HetChiSq** | **HetDf** | **HetPVal** |
| Intergenic | 2q24.1-31.1 | rs10172929 |  |  |  | 13.034 | 3 | 0.004563 |
| Intergenic | 2q24.1-31.1 | rs10201330 |  |  |  | 10.61 | 3 | 0.01403 |
| *CHRM2* | 7q33 | rs10271552 |  |  |  | 3.034 | 3 | 0.3864 |
| Intergenic | 2q24.1-31.1 | rs11896469 |  |  |  | 9.254 | 3 | 0.0261 |
| *ATXN1* | 6p25-21.2 | rs12204969 |  |  |  | 22.261 | 3 | 5.76E-05 |
| *BDNF* | 11p14 | rs12273363 | 2.328 | 1 | 0.127 | 6.973 | 4 | 0.1373 |
| *BDNF* | 11p14 | rs12288512 | 2.399 | 1 | 0.1214 | 7.192 | 4 | 0.1261 |
| *LY75* | 2q24.1-31.1 | rs16844374 |  |  |  |  |  |  |
| *ATXN1* | 6p25-21.2 | rs17606174 | 0.136 | 1 | 0.7123 | 20.672 | 4 | 0.000368 |
| *ATXN1* | 6p25-21.2 | rs17606216 |  |  |  | 21.996 | 3 | 6.54E-05 |
| *IGF2R* | 6q26 | rs1805075 |  |  |  | 5.637 | 3 | 0.1307 |
| Intergenic | 14q11.2-12 | rs1872159 | 0.669 | 1 | 0.4132 | 9.255 | 4 | 0.05504 |
| Intergenic | 6p25-21.2 | rs195371 |  |  |  | 16.831 | 3 | 0.000766 |
| *TRIM31* | 6p25-21.2 | rs2023472 | 0.742 | 1 | 0.3889 | 18.243 | 4 | 0.001106 |
| *DTNBP1* | 6p23 | rs2619545 | 3.324 | 1 | 0.06826 | 6.794 | 4 | 0.1472 |
| *ALDH5A1* | 6p23 | rs2760138 |  |  |  |  |  |  |
| Intergenic | 14q11.2-12 | rs2807822 | 4.077 | 1 | 0.04348 | 17.763 | 4 | 0.001373 |
| Intergenic | 14q11.2-12 | rs3811222 | 1.655 | 1 | 0.1982 | 9.737 | 4 | 0.0451 |
| Intergenic | 2q24.1-31.1 | rs4289149 | 7.551 | 1 | 0.005998 | 13.734 | 4 | 0.008196 |
| *DRD2* | 11q23 | rs4630328 |  |  |  | 6.126 | 3 | 0.1056 |
| Intergenic | 2q24.1-31.1 | rs4972741 |  |  |  | 12.511 | 3 | 0.005822 |
| *CHRM2* | 7q33 | rs6467694 | 0.543 | 1 | 0.4611 | 10.805 | 4 | 0.02884 |
| *DRD2* | 11q23 | rs6589377 | 1.127 | 1 | 0.2885 | 6.344 | 4 | 0.1749 |
| Intergenic | 2q24.1-31.1 | rs6721348 |  |  |  | 12.302 | 3 | 0.006418 |
| Intergenic | 6p25-21.2 | rs6929774 | 7.189 | 1 | 0.007335 | 17.342 | 4 | 0.001659 |
| *C6orf227* | 6p25-21.2 | rs6929819 | 7.124 | 1 | 0.007606 | 17.253 | 4 | 0.001726 |
| Intergenic | 14q11.2-12 | rs7149201 | 4.41 | 1 | 0.03573 | 10.292 | 4 | 0.03578 |
| *DTNBP1* | 6p23 | rs760666 | 1.958 | 1 | 0.1617 | 4.851 | 4 | 0.303 |
| Intergenic | 14q11.2-12 | rs762578 | 0.645 | 1 | 0.4219 | 9.944 | 4 | 0.04139 |
| *DTNBP1* | 6p23 | rs7758659 | 1.954 | 1 | 0.1622 | 4.834 | 4 | 0.3047 |
| *IGF2R* | 6q26 | rs8191818 | 0.071 | 1 | 0.7902 | 5.932 | 4 | 0.2043 |
| *IGF2R* | 6q26 | rs8191821 | 0.073 | 1 | 0.7869 | 5.963 | 4 | 0.2019 |
| *IGF2R* | 6q26 | rs8191898 | 0.079 | 1 | 0.7786 | 6.294 | 4 | 0.1783 |
| *DTNBP1* | 6p23 | rs875462 | 2.246 | 1 | 0.134 | 4.642 | 4 | 0.326 |
| Intergenic | 14q11.2-12 | rs877726 | 1.309 | 1 | 0.2526 | 10.634 | 4 | 0.031 |
| *DTNBP1* | 6p23 | rs9296983 | 1.945 | 1 | 0.1631 | 4.888 | 4 | 0.299 |
| *IGF2R* | 6q26 | rs9457827 | 0.078 | 1 | 0.7799 | 6.262 | 4 | 0.1804 |
| Noncoding gene | 6p25-21.2 | rs993600 | 1.182 | 1 | 0.277 | 11.019 | 4 | 0.02635 |
| Intergenic | 2q24.1-31.1 | rs995711 | 0.495 | 1 | 0.4817 | 10.277 | 4 | 0.036 |
